# Automatic Screening for Perturbations in Boolean Networks

**DOI:** 10.3389/fphys.2018.00431

**Published:** 2018-04-24

**Authors:** Julian D. Schwab, Hans A. Kestler

**Affiliations:** ^1^Medical Faculty, Institute of Medical Systems Biology Ulm University, Ulm, Germany; ^2^International Graduate School of Molecular Medicine Ulm University, Ulm, Germany

**Keywords:** systems biology, regulatory networks, Boolean networks, dynamic model, simulation, perturbation studies, SAT solving

## Abstract

A common approach to address biological questions in systems biology is to simulate regulatory mechanisms using dynamic models. Among others, Boolean networks can be used to model the dynamics of regulatory processes in biology. Boolean network models allow simulating the qualitative behavior of the modeled processes. A central objective in the simulation of Boolean networks is the computation of their long-term behavior—so-called attractors. These attractors are of special interest as they can often be linked to biologically relevant behaviors. Changing internal and external conditions can influence the long-term behavior of the Boolean network model. Perturbation of a Boolean network by stripping a component of the system or simulating a surplus of another element can lead to different attractors. Apparently, the number of possible perturbations and combinations of perturbations increases exponentially with the size of the network. Manually screening a set of possible components for combinations that have a desired effect on the long-term behavior can be very time consuming if not impossible. We developed a method to automatically screen for perturbations that lead to a user-specified change in the network's functioning. This method is implemented in the visual simulation framework ViSiBool utilizing satisfiability (SAT) solvers for fast exhaustive attractor search.

## 1. Introduction

Internal and external conditions cause a biological system to change its behavior over time. Mathematical models have become invaluable tools to gain insights into the complex dynamics of biological systems. Boolean networks are one kind of dynamic models based on two-valued logic. Boolean networks can be modeled manually by extraction of Boolean functions from literature resources or inferred automatically from time-series data (Lähdesmäki et al., 2003; Maucher et al., 2011, 2012; Hopfensitz et al., 2012). Simulation of Boolean networks allows for studying various dynamic network properties of the investigated systems. The long-term behavior of the modeled system often corresponds to biologically relevant phenotypes (Naldi et al., 2015). Furthermore, the dynamics of Boolean networks can aid in identifying components that are crucial for these phenotypes. For instance, the effects of depriving or over-representing one element in the system can be measured in the form of changes in the long-term behavior. However, the number of possible perturbations increases rapidly with a larger model size. We developed a method to automatically screen for perturbations that cause a desired effect on the long-term behavior of the system.

There are various tools and frameworks to model, simulate or visualize different types of Boolean networks. The R-package BoolNet comprises a number of simulation algorithms, for instance, attractor search, network perturbation or robustness analysis for synchronous, asynchronous, and probabilistic Boolean networks (Müssel et al., 2010). Additionally, it allows for visualization of dependencies in the network and attractors. However, BoolNet requires programming skills and a basic understanding of the programming language R.

GUI-based software like GinSim (Gonzalez et al., 2006) incorporates different simulation methods for logical models without temporal predicates, including the simulation of manually specified perturbations.

MaBoSS (Stoll et al., 2017) is a tool to simulate Boolean networks stochastically. MaBoSS focuses on a vast number of simulation methods including perturbation studies without the ability to model. We chose to include our methods to automatically screen for perturbations into the existing Java-based framework ViSiBooL (Schwab et al., 2017a). ViSiBooL extends the Boolean network paradigm by temporal predicates and is a light-weight stand-alone modeling and simulation framework. It specifically aims at a straight-forward and easy-to-use modeling and simulation functionality also used by life scientists without any programming skills.

The framework allows to model Boolean networks from scratch and to load existing network models from different sources. Boolean networks can be modeled via graph representations and text-based. The supported SBML-qual standard (Chaouiya et al., 2013) and a simple text network specification format allow for tight interoperability with other common software tools.

In the following we will first briefly define Boolean networks, show how SAT solving (Schöning and Torán, 2013) can be used for attractor search, and then outline our automated screening procedure which can also use temporal predicates in Boolean networks. Finally, we will give some simulation results on a model of the senescence-associated secretory phenotype (SASP).

## 2. Methods

### 2.1. Boolean networks

Boolean networks are a class of simple logical models that can be used for the modeling of dynamic biological processes such as gene regulation (Kauffman, 1969, 1994). Each component of the modeled system is described by a Boolean variable. It can either be active (true/1) or not (false/0). Dependencies between the different components in the network are described by Boolean functions. The state of a Boolean network with *n* components at time *t* is described by a Boolean vector **x**(*t*) = (*x*_1_(*t*), …, *x*_*n*_(*t*)). The value of each component *x*_*i*_ at a time *t* is determined by its corresponding transition function $f_{i}:{𝔹}^{n}\to 𝔹$. The successor state **x**(*t* + 1) is calculated as follows : **x**(*t* + 1) = (*f*_1_(**x**(*t*), …, *f*_*n*_(**x**(*t*)). Here, an exemplary Boolean network with three components *x*_1_, *x*_2_, *x*_3_ and their transition functions is defined : *f*_1_(**x**(*t*)) = ¬*x*_1_(*t*), *f*_2_(**x**(*t*) = *x*_1_(*t*)∨*x*_2_(*t*)), *f*_3_(**x**(*t*) = *x*_1_(*t*) ∧ ¬*x*_2_(*t*)). There are three major types of Boolean networks -synchronous, asynchronous and probabilistic. In synchronous Boolean networks all variables are updated at the same time. In asynchronous Boolean networks only one randomly chosen variable is updated at each time step **x**(*t* + 1) = (*x*_1_, …, *f*_*i*_(**x**(*t*)), …, *x*_*n*_), where *i*∈[1, *n*] (Harvey and Bossomaier, 1997).

Probabilistic Boolean networks allow for specifying more than one transition function per variable in the network. Each of these functions has a probability of being chosen, where the probabilities of all functions for one variable sum up to 1 (Shmulevich et al., 2002).

The methods presented in the following focus on the simulation of synchronous Boolean networks.

The dynamics of the Boolean networks are studied via examining the transitions from one state to another. The number of states in Boolean networks is finite (2^*n*^ in a network with *n* components). Consequently, the network eventually converges to a recurring number of states after a number of state transitions. These cycles of states are called attractors and represent the long-term behavior of the Boolean network. As already previously mentioned, attractors are of special interest as they often represent biologically relevant behaviors (Naldi et al., 2015). This could be shown in a number of publications successfully using Boolean networks to model the qualitative behavior of a variety of tissues in different organisms (Albert and Othmer, 2003; Fauré et al., 2006; Herrmann et al., 2012; Dahlhaus et al., 2016; Linke et al., 2017; Meyer et al., 2017). All states leading to the same attractor are associated to its so-called basin of attraction (Saadatpour and Albert, 2013). All basins of attraction together comprise the complete number of states.

### 2.2. Attractor search and SAT

There are different types of algorithms for attractor search in Boolean networks. Basic algorithms for exhaustive attractor search examine each state. However, these algorithms are demanding in terms of runtime ($O\left(2^{n}\right)$) and memory ($O\left(2^{n}\right)$) (Hopfensitz et al., 2013). A number of other algorithms to search for attractors have been proposed. Some of them search efficiently for attractors of length one (Akutsu et al., 2011; Veliz-Cuba et al., 2014). An algorithm that searches for attractors of different length very efficiently is based on SAT-solving (Dubrova and Teslenko, 2011; Naldi et al., 2015). Especially for networks with modest connectivity, this algorithm is more efficient than the exhaustive algorithms that examine every possible state.

Solving a satisfiability (SAT) problem, is basically finding an assignment that satisfies a Boolean formula, i.e., the Boolean formula returns true (Schöning and Torán, 2013). The SAT-solving approach can now be adapted to perturbation studies and the temporal extension in Boolean networks. In the following a basic SAT-based attractor search algorithm is briefly described.

Formally, a state transition can be defined as follows: $T\left(\text{x}\left(t\right),\text{x}\left(t+1\right)\right)=\wedge_{i=1}^{n}x_{i}\left(t+1\right)↔f_{i}\left(x_{1}\left(t\right),\ldots ,x_{n}\left(t\right)\right)$, where *n* is the number of components in the network. In the algorithm a path—a consecutive sequence of states—is represented by such a Boolean formula. A path of length two in the previously given example network is defined as follows : *T*(**x**(*t*), **x**(*t* + 1)) = (*x*_1_(*t* + 1) ↔ ¬*x*_1_(*t*))∧(*x*_2_(*t* + 1) ↔ (*x*_1_(*t*) ∨ *x*_2_(*t*)))∧(*x*_3_(*t* + 1) ↔ *x*_1_(*t*)∧¬*x*_2_(*t*)). A satisfying assignment for this formula corresponds to a valid, existing path. A SAT-solver can now be used to find all satisfying assignments—each corresponds to one path through the state graph of the Boolean network. Attractors are deduced from these valid paths. Starting with an initial length all valid paths in the Boolean network are determined. First, to compute the the valid solutions for a path the transition formula has to be unfolded. The resulting conjunction of clauses is then solved using a SAT solver.

Next, to detect attractors it is checked whether a state occurs more than once in the path. Obviously, all states between two equal states belong to the attractor. If an attractor is in the path, it is stored and its states are added to the formula as constraints. All other paths including the same attractor are no valid solution anymore. Consequently, the whole basin of attraction of the found attractor is excluded from the search space. If the found path is attractor free, the analyzed sequence of states has to be prolonged to reach the attractor. This procedure is repeated until there is no other valid solution found by the SAT-solver. This means all valid paths to attractors were examined and all existing attractors are found.

In our implementation we used the SAT-solver MINISAT (Eén and Sörensson, 2004) which is based on the idea of conflict-driven backtracking (Marques-Silva and Sakallah, 1999).

### 2.3. Temporal predicates in attractor search

In synchronous Boolean networks all components are updated at the same time and their value is determined according to the previous state of the network. These assumptions can restrict the modeling or may require hypothetical delay nodes. Biological processes happen on different time scales. In some processes the accumulation of a product over several time steps is required to activate the production of another component. Different components might have different latency periods. The temporal predicates allow the modeling of such latency periods (Schwab et al., 2017a).

In this temporal extension the next state **x**(*t* + 1) may not only depend on the previous time step **x**(*t*), but also any other predecessor state **x**(*t*−Δ), Δ = {1, 2, …, *t* − 1}.

For this extension a history of previous values of the relevant components are stored in addition to the current values of the network at time *t*.

This temporal extension to the synchronous Boolean network model includes two temporal operators. One that allows a direct specification of operations like an accumulation of a gene product over a number of time steps. This operator ALL only evaluates true if a specified term is valid for a given number of time steps. The second operator ANY evaluates true if a term is valid at least once in a specified period of time. The previously described SAT based attractor search is now expanded to include these operators. To find a solution for the unfolded formula of a path each network component at each time step is mapped to another variable. Exemplarily, a path from *t* to *t* + 1 in a network with three components *x*_1_, *x*_2_, *x*_3_ is mapped to six variables *v*_1_, …, *v*_6_, where *x*_1_(*t*) = *v*_1_, *x*_2_(*t*) = *v*_2_, …, *x*_1_(*t* + 1) = *v*_4_, …. Consequently, the formula for the SAT-solver consists of *l* · *n* variables, where *n* is the number of components and *l* the length of the path. In these temporal Boolean networks the value of a network component does not only depend on the values of the previous state. To enable exhaustive attractor search the mapping had to be changed to reference back to values before the previous time step.

The temporal extension allows the network to stay in a state for more than one time step before moving to another. This prevents searching for multiple occurrences of a state in the path to detect attractors. Not only the states in the path are compared but also their history. True equality of states to detect attractors is only given if their history is also equal.

### 2.4. Screening for meaningful perturbations

Boolean networks can be used for the simulation of various perturbations. Components can be stripped from the system (called knock-down here) or the system can have a surplus of some component (called over-expression here). These behaviors of component *x*_*i*_ can be formally described by

$$x_{i}(t+1)=\{\begin{array}{ll} 0\; & x_{i}\text{ is knocked down},\\ 1\; & x_{i}\text{ is overexpressed,}\\ f_{i}(x(t))\; & \text{else}. \end{array}$$

Such interventions of the system may have major effects on its dynamic behavior. The new framework implements various features to investigate the effects of such perturbations.

#### 2.4.1. Single path perturbation

Local attractor search from a user-specified initial condition can be modified by knock-down or over-expression of components of interest. The resulting attractor is instantly computed and visualized, which allows for fast comparison of original and perturbation behavior.

#### 2.4.2. Global network perturbation

Global effects of perturbations are determined via an extension of the exhaustive search algorithm described in the previous section. Our SAT-solving algorithm was extended to support also fixed components. This implies that in certain cases the Boolean formulae can be simplified. In our procedure this is being performed on a symbolic level prior to conversion into a conjunctive normal form (CNF) for SAT solving.

#### 2.4.3. Automated screening for meaningful perturbations

The two previous methods both rely on user-specified perturbations. However, there are cases in which a user aims at investigating which perturbation shows a wanted effect. For this reason another method was developed.

**Figure 1:**
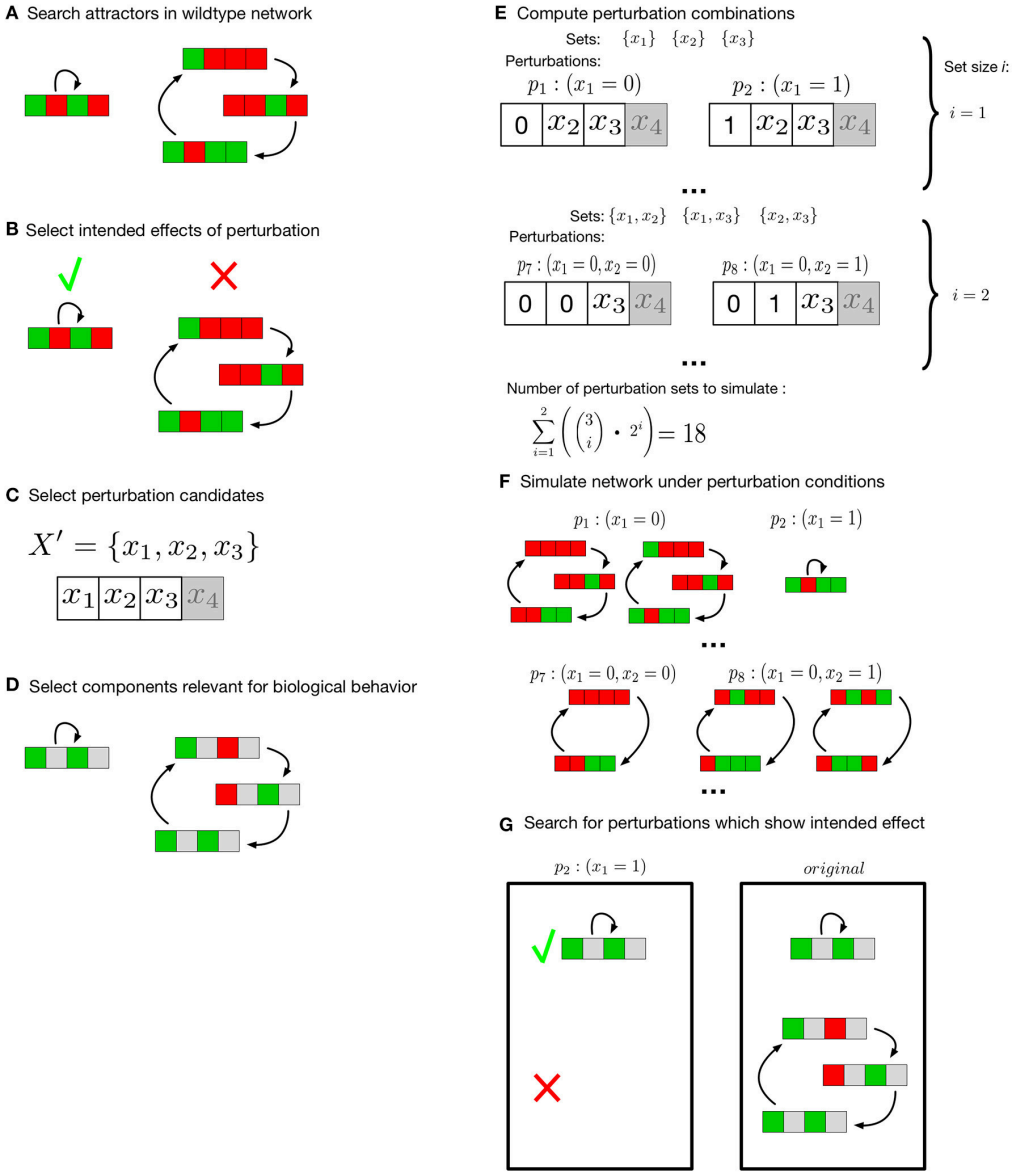
Search for meaningful perturbation effects. Colors green and red represent the Boolean values *true* and *false*, components the user declared irrelevant for the analysis are gray. **(A)** attractors of the unperturbed network are searched exhaustively. **(B)** The user specifies the effects intended by perturbation of the network. **(C)** Components to evaluate under perturbation conditions are selected. **(D)** Selection of components of interest for the attractors under investigation. After the setup by the user **(B–D)** all possible combinations of perturbations are computed **(E)**. Attractors for all perturbation sets are computed **(F)** and compared to the original networks **(G)**. All perturbation sets that match the intended effects are returned.

Here, the user can specify a set of perturbation candidates (Figure 1C). Among these candidates, the method searches for all perturbations and combinations of perturbations which show a desired effect.

This effect is also user-defined. Attractors which are intended to exist or not exist under perturbation conditions can be selected (Figure 1A,B). For *k* user-selected components of interest (Figure 1C) all knock-down and/or over-expression combinations of size one up to a user-specified maximum size m are generated. This results in a set *P* of perturbation combinations to test. Each perturbation *p*_*i*_∈*P* is another combination of a number of components in one of the possible perturbation types (knock-down/over-expression). For instance, a set of components $X^{\prime }=\left\{x_{1},x_{2},x_{4}\right\}$ is selected and the maximum combination size is two. This results in *P* = {(*x*_1_ = 0), (*x*_1_ = 1), …, (*x*_1_ = 0, *x*_2_ = 0), (*x*_1_ = 0, *x*_2_ = 1), …}, |*P*| = 18 (Figure 1E). Next, these $\sum_{i=1}^{m}\left(\begin{array}{c} k\\ i \end{array}\right)\cdot 2^{i}$ combinations of perturbations are evaluated (Figure 1F). In this evaluation the longterm behavior of the perturbed network is compared to the longterm behavior of the unperturbed network model (Figure 1G). Not all components of the network might be of interest for every description of a biologically relevant behavior. Thus, the user can specify a set of components and the resulting attractors of perturbed network and original network are compared on the basis of these components (Figure 1D). Finally, all perturbation combinations pi that match the intended longterm behavior are returned by the algorithm. To increase the simulation speed in our implementation, the different perturbation combinations are evaluated in parallel. The number of parallel instances scales with the number of available cores.

### 2.5. Biological example

To illustrate the feasibility of the methods we used the Boolean network described in Meyer et al., 2017, which is a model for the SASP after DNA damage induced senescence. Cellular senescence is a tumor suppressor mechanism which arrests cells before becoming malignant (Coppé et al., 2010; Muñoz-Espín and Serrano, 2014). Senescent cells secrete different factors to attract phagocytic immune cells. Early SASP is probably beneficial to clear the damaged cells. However, once the immune system cannot keep up with the emergence of damaged cells, counteracting the SASP can prevent tissue damage (Meyer et al., 2017). SASP can, for instance, turn senescent fibroblasts into pro-inflammatory cells with the ability to promote tumor progression (Coppé et al., 2010).

**Figure 2:**
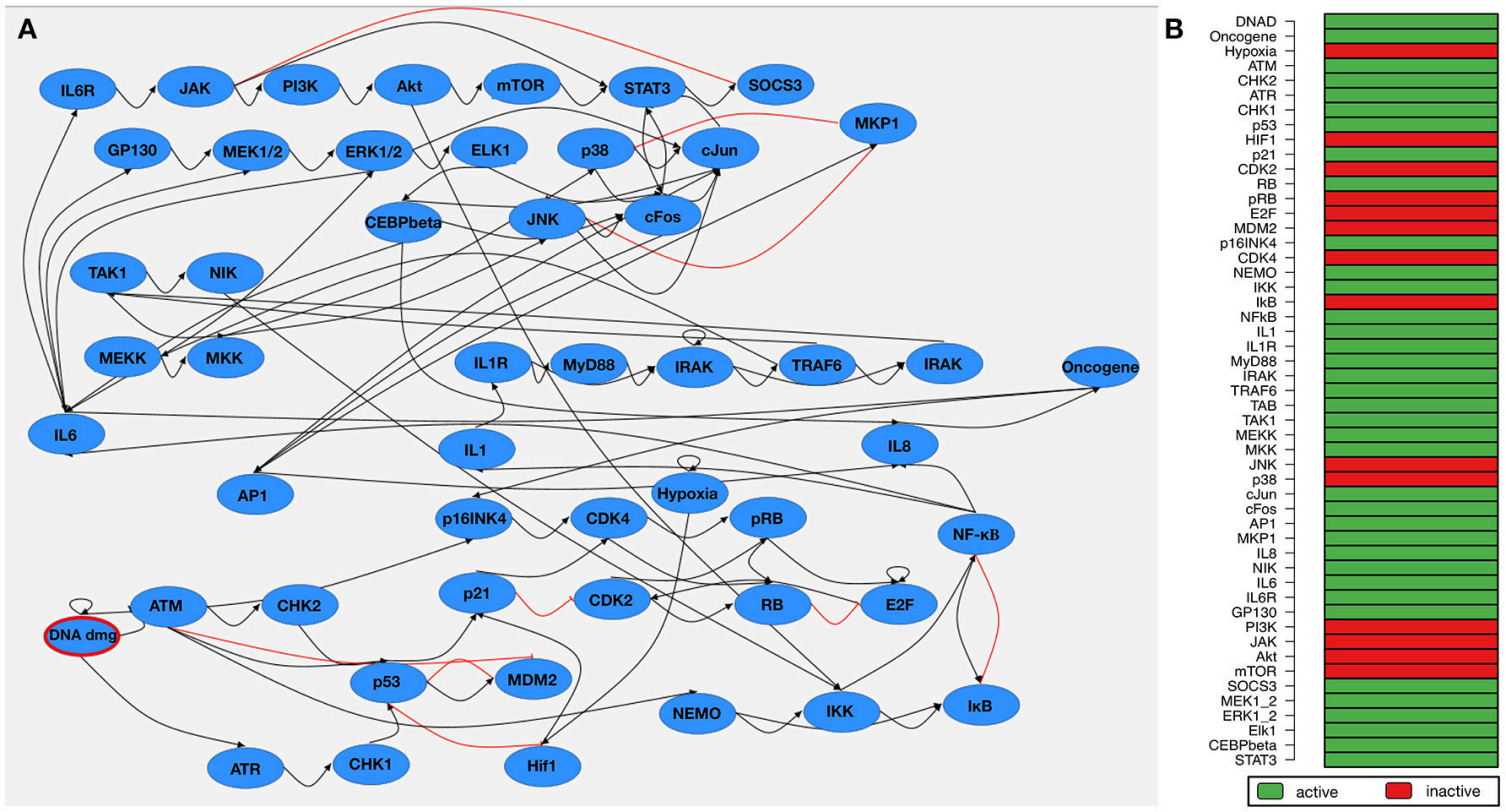
Boolean network of the senescence-associated secretory phenotype (SASP). **(A)** Network wiring of the Boolean network. Blue nodes depict the components of the network model. Black (Red) edges represent a activatory (inhibitory) dependency between the connected components. DNA damage as input node of the network is marked in red. **(B)** Steady-state attractor of the SASP-network under DNA damage conditions (DNA damage input is 1/true). A green (red) row indicates that the corresponding component is active (inactive) in the attractor.

The published Boolean network model comprises of two interacting subnetworks—one for DNA damage signaling and one modeling the inflammatory response. The complete model contains 51 components (Figure 2A). Attractor search simulation of the network model shows an active immune response after DNA damage (Figure 2B). Mayer et al. used the Boolean network model to hypothesize about perturbations that prevent an immune response after DNA damage. These perturbations aim at counteracting the SASP to give the immune system time to catch up. Manual perturbation simulations of the network identified knocking-out NF-κB Essential Modulator (NEMO) is a promising candidate to prevent an immune response—a hypothesis which could be validated by *in-vitro* approaches (Meyer et al., 2017).

## 3. Simulation results

Evaluation was performed with the previously described Boolean network model of the SASP. In Meyer et al. (2017) different perturbation candidates were manually tested for their deactivation of the major SASP-mediators after DNA damage. Also, attractors had to be analyzed manually to examine feasible candidate perturbations. This approach can be very time consuming for a growing number of candidates to test.

For the evaluation here, we screened the Boolean network model for perturbation candidates that inhibit an immune response after DNA damage. The results were then compared to the results manually investigated by Meyer et al.

We selected IL-1, IL-6, and IL-8 as components in the Boolean network which are overlapping with the up-regulated factors in the SASP according to Coppé et al., 2010. This correlates with the results of the exhaustive attractor search in the Boolean network under DNA damage conditions (DNA damage input is on, Hypoxia is off, Figure 2B shows IL1, IL6, and IL8 are active in the attractors).

**Figure 3:**
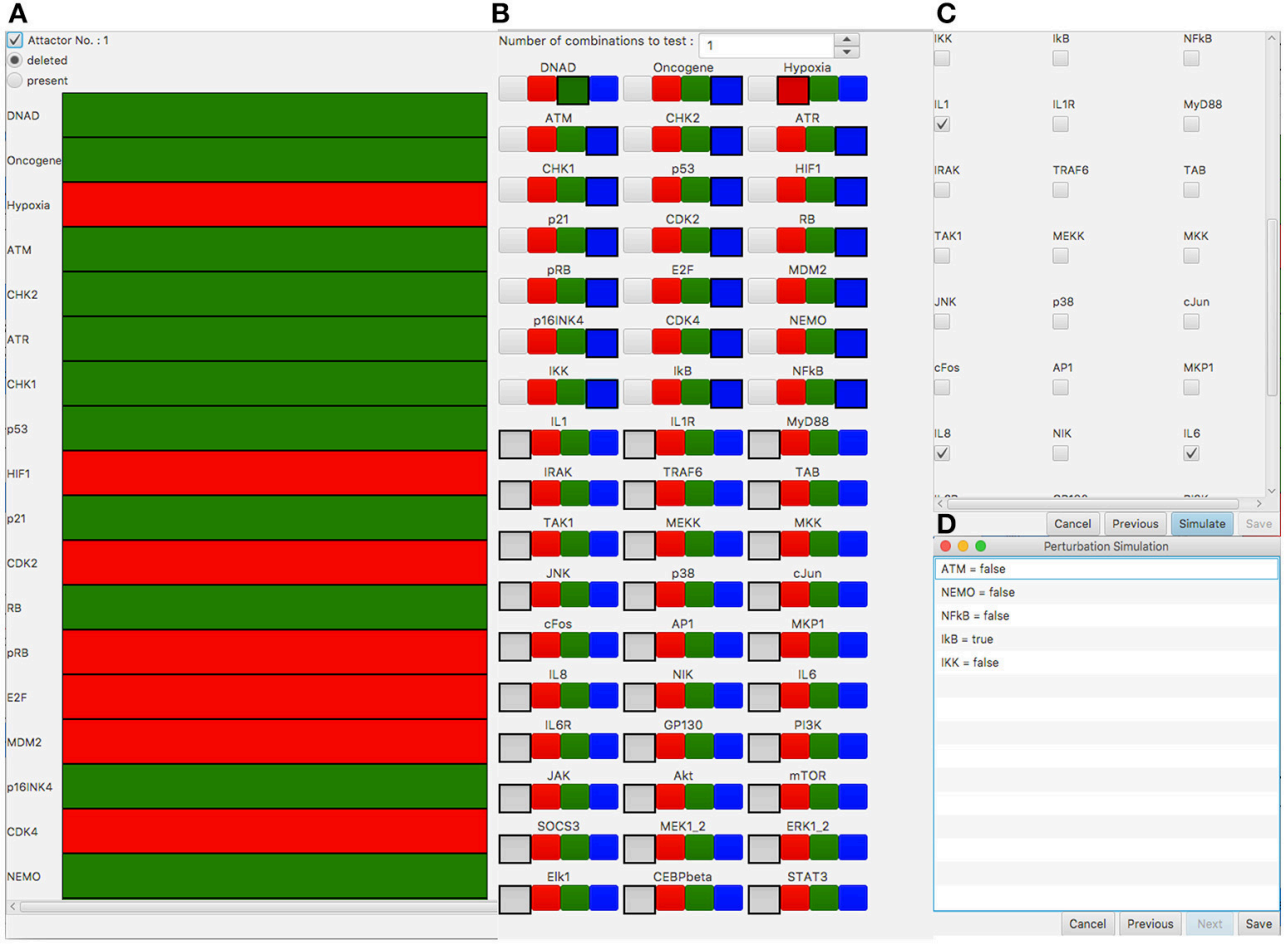
Perturbation screening results in the SASP model (Meyer et al., 2017). **(A)** Attractor under DNA damage conditions which should be removed by perturbation. Green (Red) rows indicate the corresponding component is active (inactive) in the attractor. **(B)** Selection of perturbation candidates to test. Selection of the gray box indicates the component is not of interest for perturbation. Green (Red) means the component is over-expressed (knocked-out). Blue means that both possible perturbations (over-expression/knock-out) are tested for the corresponding component. In this simulation all 19 non-input components that belong to the DNA damage signaling subnetwork of the SASP network are perturbation candidates for knock-out or over-expression. The inputs DNA damage (DNAD) and Hypoxia are fixed to over-expression and knock-out, respectively. The inflammatory signaling part of the network is not selected for perturbation. **(C)** Selection of the genes of interest for attractor comparison. IL1, IL6, and IL8 are selected, which means these components have to be inactive after perturbation. **(D)** Results showing the perturbation candidates that removed the attractor in **(A)** according to the components selected in **(C)**.

For the automatic screening, we selected to remove the attractor (Figure 3A) according to their state of the interleukins IL1, IL6, and IL8 (Figure 3C). With the perturbation, we aim at blocking the inflammatory response after DNA damage but not at a general inhibition of pro-inflammatory signaling. Thus, we chose each single-component perturbation of all components of the DNA damage signaling subnetwork of the network model as perturbation candidates (19 components which lead to 38 perturbations to test, see Figure 3B). During the screening process, attractor search is performed for each candidate perturbation. The attractors are then compared to the original attractors of the network under DNA damage conditions. Perturbations which result in attractors that are differing from the original ones according to there values of a selected set of components (here IL1, IL6, IL8) are returned as valid perturbations.

The screening took 64 s on a MacBook Pro (Intel Core I5, 3.1 GHz and 16GB RAM). The analysis shows a deactivation of the immune response for a knock-out of NEMO, NF-κB, ATM, IKK, or an over-expression of IκB (Figure 3C). In addition to the suggested NEMO knock-out of Meyer et al. (2017), the automatic screening reveals four new candidate perturbations - knock-out of ATM, NF-κB, and IKK as well as over-expression of IκB. One possible explanation is their ability to act as SASP-triggering factors, which are mainly relayed through NF-κB. NF-κB has a direct regulatory link to IL1, IL6, and IL8. IKK and IκB both have a direct effect on NF-κB and thus have a regulatory impact on the different Interleukins. NEMO has a regulatory effect on these components via IKK and NF-κB and ATM via NEMO/IKK/NF-κB. The shortest paths from the perturbed components to the Interleukins IL1, IL6, IL8 are between one (perturbation of NF-κB) and four (perturbation of ATM) interactions long. This shows the ability to not only identify direct but also indirect regulators as meaningful perturbation candidates in this complex network by our automatic procedure.

## 4. Conclusion

Perturbation studies of Boolean networks can provide more detailed information about the network's inner dynamics. Among others, network perturbation can help to identify therapeutic targets (Saadatpour et al., 2011), to measure a network's capability to compensate mutations (Kwon et al., 2016) or to quantify the robustness of Boolean networks (Schwab et al., 2017b). Furthermore, perturbation of components can be a helpful, assistive tool to check for the expected behavior during the modeling process. Simulation of network perturbation is commonly used in multiple frameworks (Gonzalez et al., 2006; Müssel et al., 2010; Stoll et al., 2017).

The automated screening for perturbations that fulfill user-defined changes in the long-term behavior is—to our best knowledge—a new feature for the analysis of Boolean networks. This feature aims at identifying crucial components for developing a specific long-term behavior. Finding perturbations that eliminate a specified long-term behavior can also be used to screen for therapeutic targets.

These methods were integrated into the Java framework ViSiBooL (Schwab et al., 2017a). ViSiBooL aims at a straight-forward and easy-to-use modeling and simulation of Boolean networks. The temporal extension of synchronous Boolean networks allows for a more realistic way of modeling biological processes while maintaining the simple interpretation of synchronous Boolean networks. Moreover, the temporal operators ALL and ANY provide a straight-forward methodology to simplify large terms to model processes over more than one time step. All implemented network perturbation experiments support the temporal network extensions.

## Availability

The software is available from http://sysbio.uni-ulm.de/?Software:AutoScreenBN

## Author contributions

JS and HK: Conceived the software; JS: Implemented the software; JS and HK: Wrote the paper; HK: Supervised the project; HK: Obtained funding for the project.

### Conflict of interest statement

The authors declare that the research was conducted in the absence of any commercial or financial relationships that could be construed as a potential conflict of interest.
